# Olive-Derived Nutritional Interventions in Exercise: Effects on Performance, Fatigue, Recovery, and Muscle Adaptation—A Systematic Review

**DOI:** 10.3390/nu18213699

**Published:** 2026-11-07

**Authors:** Laura Gil-Caselles

**Affiliations:** Research Group HUMSE, Faculty of Sports Sciences, University of Murcia, 30720 Murcia, Spain; laura.gilc@um.es; Tel.: +34-868887580

**Keywords:** extra virgin olive oil, olive polyphenols, maslinic acid, exercise performance, recovery

## Abstract

**Background/Objectives:** Olive-derived foods and bioactive compounds are increasingly investigated in sports nutrition, but their effects on exercise outcomes remain unclear. This systematic review evaluated randomized controlled evidence on olive-derived nutritional interventions in humans. **Methods:** MEDLINE/PubMed, Scopus, Web of Science Core Collection, SPORTDiscus, the Cochrane Central Register of Controlled Trials (CENTRAL), ClinicalTrials.gov, and the World Health Organization International Clinical Trials Registry Platform (WHO ICTRP) were searched from inception to 30 September 2026. Randomized controlled trials of orally administered olive-derived interventions in healthy participants undergoing exercise, training, or physical-function assessment were included. Risk of bias was assessed using the revised Cochrane Risk of Bias tool for randomized trials (RoB 2), with two assessments rated as low risk and eight as having some concerns, and findings were synthesized without meta-analysis because of substantial heterogeneity. **Results:** Eleven reports representing 10 independent trials evaluated chemically distinct interventions: extra virgin olive oil, hydroxytyrosol-rich olive-fruit-water preparations, oleuropein-rich olive-leaf extracts, and maslinic acid. In specific exercise settings, maslinic acid was associated with lower perceived fatigue or muscle soreness, while selected muscle-related outcomes differed in trials involving older adults. Other preparations yielded heterogeneous physiological, metabolic, or fatigue-related findings, without consistent improvements in objective exercise performance. **Conclusions:** The available evidence does not establish a common ergogenic effect across olive-derived products. Any potential benefit must be interpreted in relation to the specific formulation, dose, population, exercise context, and outcome assessed. Independent confirmation is required before practical recommendations can be established.

## 1. Introduction

Physical exercise induces metabolic, mechanical, and inflammatory responses that support training adaptation but may also contribute to transient fatigue, muscle soreness, and impaired muscle function [1,2]. Redox and inflammatory signaling are integral to skeletal-muscle remodeling, so changes in exercise-induced biomarkers should not automatically be interpreted as beneficial [1,2]. Alongside adequate energy and nutrient intake, interest has grown in polyphenol-rich foods and other plant-derived compounds as potential modifiers of exercise-related physiological responses and recovery [3,4,5].

The olive tree, *Olea europaea* L. (Oleaceae), is an evergreen tree or shrub native to the Mediterranean region, characterized by opposite, leathery leaves and fleshy drupes containing a central stone [6]. Its fruit and leaves are sources of chemically distinct nutritional products. Extra virgin olive oil (EVOO) is a complex lipid-rich matrix, with the lipid fraction accounting for approximately 98–99% of its composition. Oleic acid is the predominant fatty acid, generally representing approximately 55–83% of total fatty acids, while linoleic acid contributes a smaller and more variable proportion [7,8,9]. The minor fraction contains phenolic compounds, tocopherols, phytosterols, and squalene. Total phenolic concentrations in EVOO vary considerably (approximately 50–1000 mg/kg, commonly 100–300 mg/kg), depending on cultivar, fruit maturity, processing, and storage [9]. Important phenolic constituents include hydroxytyrosol, tyrosol, oleuropein-derived secoiridoids, oleocanthal, and oleacein [7,8]. These compounds have been investigated for their potential roles in redox and inflammatory signaling, although their individual contributions to the biological effects of whole EVOO cannot be isolated without appropriate comparative designs. Olives and other olive-tree tissues also contain maslinic acid, a pentacyclic triterpene chemically distinct from the phenolic constituents [10].

Other olive-derived preparations differ substantially in their chemical composition and relative abundance of bioactive constituents. In an analysis of commercial olive-leaf extracts, total phenolic concentrations ranged from approximately 7.5 to 250 g/kg, with oleuropein accounting for approximately 84–90% of the quantified phenolic fraction in the analyzed extracts [11]. Other constituents included hydroxytyrosol derivatives, verbascoside, and additional secoiridoids, while triterpenic-acid concentrations varied between products. Olive-fruit-water preparations, by contrast, concentrate water-soluble phenolic compounds recovered from the aqueous fraction produced during olive processing. OliPhenolia^®^ is manufactured by separating this aqueous fraction from the oil and pomace and concentrating it through cold ceramic-membrane filtration and reverse osmosis, according to the manufacturer [12]. The sweetened formulation investigated by Roberts et al. contained approximately 11.25 g of olive extract, 12.12 g of grape-juice concentrate, and 1.62 g of lemon juice per serving, corresponding to approximately 45.0%, 48.5%, and 6.5% of the listed ingredients by weight, respectively [13]. Independent analysis of the approximately 28-mL serving identified 315.9 mg of total phenolics, including 28.8 mg of hydroxytyrosol, 22.7 mg of an oleuropein-aglycone dialdehyde, 16.0 mg of verbascoside, and 8.7 mg of oleuropein aglycone [13]. These quantitative values refer to the analyzed formulation and should not be extrapolated to other batches or olive-fruit-water products. Moreover, the presence of multiple phenolic compounds and additional formulation ingredients prevents attribution of the observed biological responses to hydroxytyrosol alone.

The rationale for reviewing these interventions together is their shared botanical origin and their evaluation against exercise-related outcomes, not an assumption of chemical or physiological equivalence. Whole-food lipid matrices, phenolic-rich fruit-water and leaf extracts, and isolated triterpenoids differ in composition, bioavailability, dose, and plausible molecular targets [7,8,10,11]. Their combined assessment within a common systematic-review framework allows comparisons of the consistency and specificity of evidence while retaining intervention-specific interpretation. Furthermore, changes in perceived fatigue, soreness, or biochemical markers must be distinguished from demonstrated improvements in physical performance.

In sports nutrition, essential amino acids and L-carnitine are among the nutritional interventions previously investigated for muscle function or physical performance [14,15]. Evidence syntheses in broader human populations suggest that EVOO and olive phenolics can influence selected oxidative and inflammatory biomarkers, but responses vary with the preparation, dose, and measured outcome [16,17,18]. Reviews of Mediterranean dietary patterns have also considered physical performance and muscle-related endpoints [19,20]; however, the effects of a multi-food dietary pattern cannot be attributed to olive-derived components alone.

Existing reviews addressing olive products in exercise have examined EVOO in athletes or focused on olive-derived polyphenols and exercise-induced inflammation [21,22]. The present review differs by restricting inclusion to randomized controlled human trials and jointly examining EVOO, olive-fruit-water preparations, olive-leaf extracts, and olive-derived triterpenoids across distinct outcome domains. This approach is intended to distinguish reproducible performance effects from isolated physiological, perceptual, or biochemical responses, without treating all olive-derived interventions as a single ergogenic strategy.

Accordingly, this systematic review evaluated randomized controlled evidence on olive-derived nutritional interventions in relation to exercise performance, exercise-induced fatigue, post-exercise recovery, and skeletal-muscle function and adaptation in humans. Secondary outcomes comprised exercise-related inflammatory, oxidative, immune, metabolic, and mitochondrial responses. The review also aimed to identify methodological limitations and priorities for future research.

## 2. Materials and Methods

### 2.1. Protocol and Reporting Framework

This systematic review was conducted and reported in accordance with the Preferred Reporting Items for Systematic Reviews and Meta-Analyses (PRISMA 2020) statement [23]. The literature-search process was additionally reported according to the PRISMA-S extension for reporting searches in systematic reviews [24]. Because quantitative pooling was not considered methodologically appropriate owing to clinical and methodological heterogeneity, the synthesis was reported according to the Synthesis Without Meta-analysis (SWiM) guideline [25]. This systematic review was not prospectively registered, and no formal review protocol was published.

### 2.2. Eligibility Criteria

Eligibility criteria were defined a priori according to the Population, Intervention, Comparator, Outcomes, and Study design (PICOS) framework.

#### 2.2.1. Population

Eligible studies included healthy human participants, including competitive athletes, recreationally active individuals, and other healthy participants undergoing structured exercise, physical training, or standardized assessments of physical or skeletal-muscle function. Studies conducted in participants recruited because of diagnosed metabolic, cardiovascular, inflammatory, neuromuscular, or other clinical conditions, or as part of a rehabilitation program, were excluded. Healthy older adults were eligible when the study context was exercise, physical function, or training adaptation rather than disease treatment.

#### 2.2.2. Intervention

Eligible interventions comprised orally administered olive-derived nutritional products or bioactive compounds from *Olea europaea* L., including extra virgin olive oil, phenolic-rich olive-oil preparations, olive-fruit or olive-leaf extracts, hydroxytyrosol- or oleuropein-rich preparations, maslinic acid, and other clearly characterized olive-derived phenolic or triterpenoid compounds. For isolated compounds also present in other botanical sources, the intervention was required to be explicitly identified as olive-derived. Multi-ingredient formulations were included only when the specific contribution of the olive-derived component could be isolated through the study design. No restrictions were applied regarding dose, supplementation duration, or timing relative to exercise.

#### 2.2.3. Comparator

Eligible comparators included placebo, matched control preparations, alternative dietary oils or fats, or other control conditions that permitted isolation of the effect of the olive-derived intervention. In randomized crossover trials, the corresponding placebo or control period was considered the comparator.

#### 2.2.4. Outcomes

Eligible studies were required to report at least one outcome related to exercise performance, exercise-induced fatigue, post-exercise recovery, skeletal-muscle function, or training adaptation. To distinguish the different dimensions of exercise response, outcomes were classified into the following categories:

(1) Exercise performance outcomes, including objective measures of exercise capacity, power output, sprint performance, time to exhaustion, and other standardized exercise tests directly reflecting physical performance.
(2) Physiological responses and exercise efficiency, including running economy, oxygen-uptake kinetics, cardiorespiratory coordination, and other physiological responses measured during or following exercise.
(3) Perceptual outcomes, including ratings of perceived exertion, subjective fatigue, and perceived recovery.
(4) Muscle soreness and recovery-related outcomes, including perceived muscle soreness and other measures of post-exercise recovery.
(5) Skeletal-muscle function and adaptation, including muscle strength, objectively assessed fatigue resistance, muscle mass, and general physical-function measures, particularly in older adults.

Secondary outcomes included exercise-related inflammatory, oxidative, immune, metabolic, mitochondrial, muscle-damage, and molecular responses that contributed to the interpretation of the primary outcomes. Adverse events and intervention tolerability were also extracted when reported.

Importantly, physiological, perceptual, biochemical, and general physical-function outcomes were distinguished from direct measures of athletic performance. Studies reporting only general clinical, cardiometabolic, or biochemical outcomes without a direct exercise-, recovery-, fatigue-, physical-function-, or skeletal-muscle-related outcome were excluded.

#### 2.2.5. Study Design

Only randomized controlled human intervention trials were included, comprising parallel-group and crossover designs, with both acute and chronic supplementation eligible. Non-randomized and quasi-experimental studies, observational designs, case reports, animal and in vitro studies, reviews, editorials, protocols without outcome data, and conference abstracts lacking sufficient information were excluded. Multiple publications arising from the same randomized trial were treated as a single independent study, although complementary outcomes reported across publications were retained.

### 2.3. Information Sources

The systematic literature search was conducted from database inception to 30 September 2026 in MEDLINE via PubMed, Scopus, Web of Science Core Collection, SPORTDiscus via EBSCOhost, and the Cochrane Central Register of Controlled Trials (CENTRAL). No restrictions were applied regarding publication year or language.

The reference lists of all included studies and relevant systematic and scoping reviews were manually screened to identify additional eligible reports. Forward citation searching of the included studies was also performed using Scopus and Web of Science.

ClinicalTrials.gov and the World Health Organization International Clinical Trials Registry Platform (WHO ICTRP) were searched to identify completed, ongoing, or potentially unpublished trials. Registry records without publicly available outcome data were not included in the intervention-effect synthesis but were considered when evaluating the completeness of the available evidence.

### 2.4. Search Strategy

The search strategy combined controlled vocabulary, where available, with free-text terms related to olive-derived nutritional interventions and exercise-related outcomes. No randomized-trial filter was applied because small nutrition and exercise trials may have been inconsistently indexed; the study design was therefore determined during screening.

The PubMed search strategy was:

(“Olive Oil”[Mesh] OR “olive oil”[tiab] OR “olive oils”[tiab] OR “extra virgin olive oil”[tiab] OR “extra-virgin olive oil”[tiab] OR EVOO[tiab] OR “Olea europaea”[tiab] OR “olive fruit”[tiab] OR “olive fruit extract”[tiab] OR “olive fruit extracts”[tiab] OR “olive fruit water”[tiab] OR “olive leaf”[tiab] OR “olive leaves”[tiab] OR “olive leaf extract”[tiab] OR “olive leaf extracts”[tiab] OR “olive-derived”[tiab] OR “olive derived”[tiab] OR “olive polyphenol”[tiab] OR “olive polyphenols”[tiab] OR “olive phenolic”[tiab] OR “olive phenolics”[tiab] OR hydroxytyrosol[tiab] OR oleuropein[tiab] OR oleocanthal[tiab] OR oleacein[tiab] OR “maslinic acid”[tiab] OR OliPhenolia[tiab])

AND

(“Exercise”[Mesh] OR “Sports”[Mesh] OR “Athletes”[Mesh] OR exercis*[tiab] OR sport*[tiab] OR athlete*[tiab] OR train*[tiab] OR “physical activity”[tiab] OR “physical performance”[tiab] OR “exercise performance”[tiab] OR endurance[tiab] OR strength[tiab] OR fatigue[tiab] OR recovery[tiab] OR “muscle soreness”[tiab] OR “muscle damage”[tiab] OR “muscle function”[tiab] OR “skeletal muscle”[tiab] OR “muscle mass”[tiab] OR “fatigue resistance”[tiab] OR “running economy”[tiab] OR “time to exhaustion”[tiab] OR “oxygen uptake”[tiab] OR VO2[tiab] OR “perceived exertion”[tiab] OR “training adaptation”[tiab])

The strategy was adapted to the indexing terms, field codes, truncation symbols, and syntax of each database. The complete search strategies for all databases and trial registries, together with the date of the final search and the number of records retrieved from each source, are provided in Supplementary Table S1.

### 2.5. Study Selection Procedure

All records retrieved from the electronic databases, trial registries, and supplementary searches were exported to Zotero (version 10.0.3). Duplicate records were identified using the reference manager and subsequently verified manually before screening. The predefined eligibility criteria were applied consistently throughout the study-selection process.

Title and abstract screening was performed by the author using the predefined eligibility criteria. Full-text eligibility was initially assessed by the author and subsequently independently verified by a second reviewer, who reassessed the full-text exclusions and checked the eligibility of the included reports. Reasons for full-text exclusion were documented and reported in the PRISMA 2020 flow diagram. Although title and abstract screening was not performed independently by two reviewers, the additional verification of full-text eligibility decisions was undertaken to reduce the risk of selection errors.

Reports originating from the same randomized trial were identified by comparing authorship, participant characteristics, intervention details, study design, and trial-registration identifiers. Specifically, the two publications by Roberts et al. [13,26] originated from the same randomized trial (NCT04959006) and were therefore considered a single independent study. Data from both reports were consolidated at the trial level, retaining complementary outcomes while ensuring that overlapping participants and outcomes were not double-counted in the synthesis.

### 2.6. Data Extraction

A standardized data-extraction form was developed and pilot-tested before formal extraction. The author extracted the relevant information from each included trial using the predefined extraction form. Extracted data included study characteristics, trial design and registration, sample size, participant demographics and training status, exercise or physical-function protocols, characteristics of the olive-derived intervention and comparator, bioactive composition, dose, supplementation duration and timing, adherence, outcome measures and assessment time points, numerical results, effect estimates and 95% confidence intervals when available, adverse events, funding sources, and author-declared conflicts of interest. The data initially extracted by the author were subsequently independently checked by a second reviewer against the original study reports, including study characteristics, intervention details, outcome measures, and reported findings. This additional verification was undertaken to reduce the risk of extraction errors and improve the reliability of the evidence synthesis.

When multiple publications reported outcomes from the same trial, data were consolidated at the trial level while retaining the source of each outcome. When relevant numerical information was incompletely reported, Supplementary Materials, trial registries, and other available study documentation were consulted. Numerical values were not inferred solely from statements of statistical significance.

### 2.7. Risk-of-Bias Assessment

Risk of bias was assessed using the revised Cochrane Risk of Bias tool for randomized trials (RoB 2) [27]. The standard RoB 2 tool was applied to parallel-group trials, whereas the specific version for crossover trials was used for randomized crossover studies. The assessment focused on the effect of assignment to the intervention.

As RoB 2 is outcome-specific, the assessment was conducted for one principal review-relevant outcome per independent trial, selected based on its direct relevance to exercise performance, fatigue, recovery, or skeletal-muscle function. The following domains were evaluated: randomization process, deviations from intended interventions, missing outcome data, measurement of the outcome, and selection of the reported result. For crossover trials, potential bias related to period and carryover effects was additionally considered.

The author performed the initial assessments, which were subsequently reviewed and corroborated by a second reviewer, with discrepancies resolved through discussion and consensus. Domain-level judgments were combined according to the RoB 2 decision rules to obtain an overall judgment for each assessed outcome, classified as low risk of bias, some concerns, or high risk of bias. One overall judgment was reported per independent trial, including trials with multiple publications, to avoid double-counting. These judgments were summarized descriptively across the 10 trials. 

Because the assessments focused on a single selected outcome per trial, the resulting judgments should not be interpreted as applying automatically to all outcomes reported within each study.

### 2.8. Data Synthesis

Study characteristics and findings were summarized in structured evidence tables. Quantitative pooling was considered inappropriate because of substantial clinical and methodological heterogeneity. Interventions differed markedly in chemical composition, including EVOO, hydroxytyrosol-rich olive-fruit-water preparations, oleuropein-rich olive-leaf extracts, and maslinic acid. Dosages ranged from acute administration of 25 mL EVOO to 30–60 mg/day maslinic acid, 100 mg/day oleuropein, or 2 × 28 mL/day of an olive-fruit-water preparation. Intervention durations varied from a single acute dose to 12 weeks of supplementation. Participants included physically active men, adolescent athletes, recreational runners, water-polo players, and healthy older adults, with substantial differences in age, sex, and training status. Exercise protocols ranged from maximal walking and aerobic running tests to resistance training, repeated sprints, water-polo training, and marathon running. Furthermore, outcomes were assessed using heterogeneous measurements, including exercise duration, running economy, oxygen-uptake kinetics, perceived exertion, muscle soreness, muscle strength, muscle mass, and biochemical markers, often at different assessment time points. These differences, together with the limited number of trials evaluating comparable intervention–outcome combinations, prevented meaningful quantitative pooling. Therefore, a structured synthesis without meta-analysis was conducted in accordance with the SWiM guideline [25].

Studies were grouped according to intervention type, including olive oil or EVOO preparations, olive-fruit or olive-fruit-water preparations, olive-leaf or oleuropein-rich extracts, and olive-derived triterpenoids, principally maslinic acid. The scientific rationale for considering these interventions within a common framework was their shared botanical origin (*Olea europaea* L.) and their investigation in relation to exercise-related outcomes, rather than an assumption of chemical or mechanistic equivalence. This framework allowed the application of consistent eligibility criteria, data-extraction procedures, and outcome classifications while preserving differences in composition, bioavailability, dose, and potential mechanisms of action. Findings were therefore synthesized by intervention type and according to the predefined outcome domains of exercise performance and physiological efficiency, fatigue and perceived exertion, post-exercise recovery and muscle soreness, skeletal-muscle function and training adaptation, and exercise-related biochemical and mechanistic responses. No common intervention effect was assumed across the different olive-derived preparations. To improve methodological transparency, a retrospective summary of the review procedures and synthesis decisions is provided in Supplementary Table S2. This document was prepared during manuscript revision in the absence of a prospectively registered protocol and should not be interpreted as a prospective protocol. Where available, reported effect sizes and 95% confidence intervals were presented alongside the corresponding outcome estimates. When these measures were not reported or could not be reliably established from the published information, available numerical results and *p*-values were retained without inferring unreported confidence intervals. Findings from very small or exploratory trials were interpreted as hypothesis-generating, and statistical significance alone was not considered sufficient evidence of a meaningful intervention effect.

### 2.9. Certainty of Evidence Assessment

The certainty of evidence for the main exercise-related outcomes was evaluated using the Grading of Recommendations Assessment, Development and Evaluation (GRADE) approach, following guidance for systematic reviews without meta-analysis [28]. This assessment was introduced during manuscript revision and was not prospectively specified.

Evidence was assessed separately according to intervention type and outcome, without assuming equivalence between different olive-derived preparations. Randomized controlled trial evidence was initially considered high certainty and evaluated across five domains: risk of bias, inconsistency, indirectness, imprecision, and publication bias.

Risk-of-bias judgments were informed by the available RoB 2 assessments and the methodological characteristics of the contributing trials. Imprecision was considered in relation to sample size, reported effect estimates, and confidence intervals where available. When only one independent trial contributed to an outcome, inconsistency could not be meaningfully assessed. Publication bias could not be formally evaluated because of the limited number of studies per comparison. The certainty of evidence was categorized as high, moderate, low, or very low, according to the GRADE framework.

## 3. Results

### 3.1. Study Selection Results

The database and trial-registry searches identified 1147 records, comprising 1126 records from bibliographic databases and 21 records from trial registries. After removal of 367 duplicate records and 4 records removed for other reasons, 776 records underwent title and abstract screening. Of these, 714 records were excluded, and 62 reports were sought for retrieval. Two reports could not be retrieved, leaving 60 reports for full-text eligibility assessment, of which 52 were excluded.

Supplementary searching identified an additional 14 records, including 12 through citation searching and 2 through other sources. One report could not be retrieved; therefore, 13 reports were assessed for eligibility, of which 10 were excluded.

**Figure 1:**
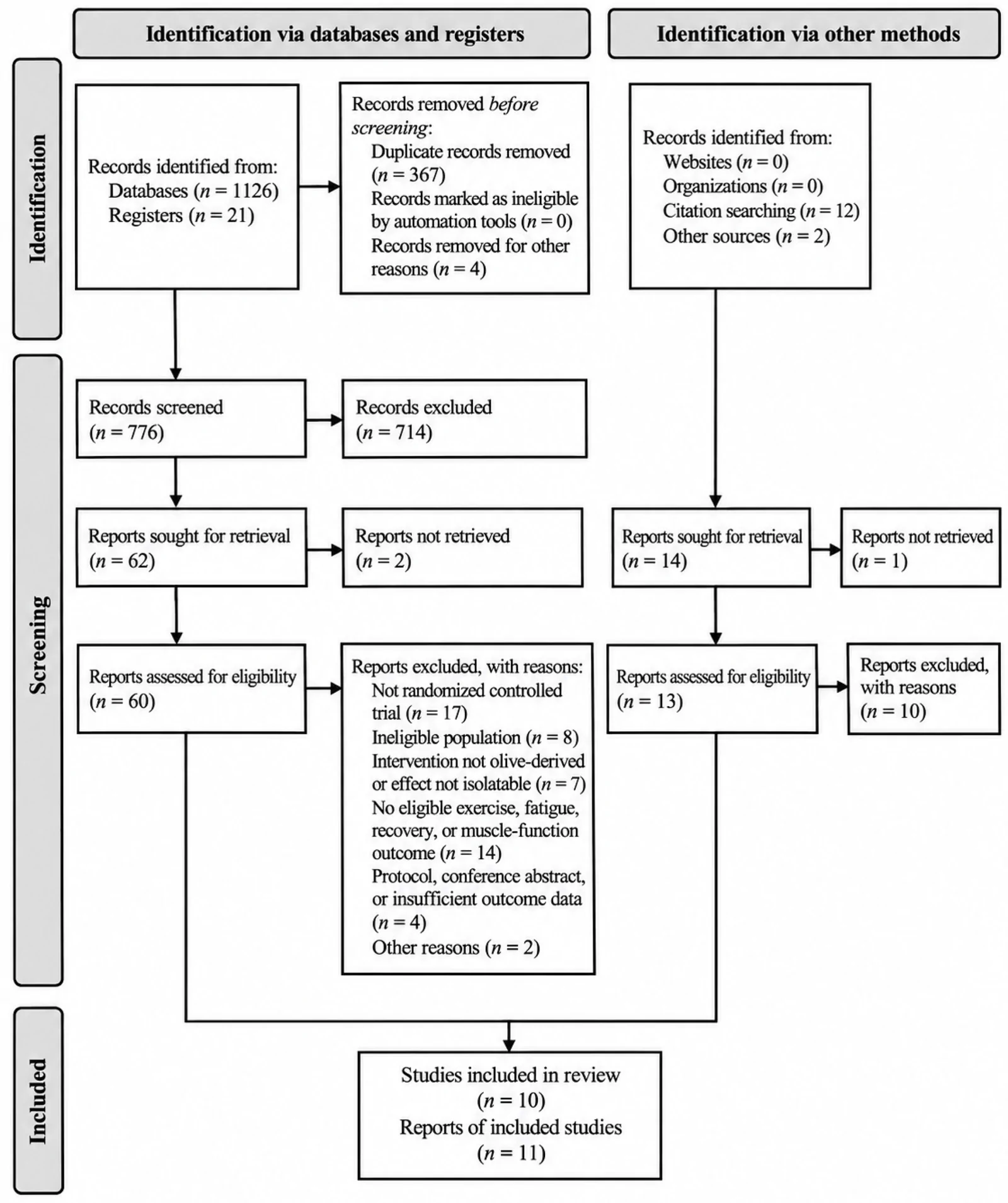
PRISMA 2020 flow diagram of the study-selection process.

Overall, 11 reports corresponding to 10 independent randomized controlled trials met the predefined eligibility criteria and were included in the systematic review. The difference reflects two publications Roberts et al. [13,26] originating from the same trial (NCT04959006), which were treated as a single independent study. The complete study-selection process and reasons for full-text exclusion are presented in the PRISMA 2020 flow diagram (Figure 1).

### 3.2. Characteristics of Included Studies

The 10 included randomized controlled trials, published between 2019 and 2026, evaluated a heterogeneous range of olive-derived nutritional interventions, including extra virgin olive oil, hydroxytyrosol-rich olive-fruit-water preparations, oleuropein-rich olive-leaf extracts, and olive-derived maslinic-acid preparations.

Study populations included competitive and recreational athletes, physically active adults, and healthy older individuals. Several trials enrolled exclusively male participants, while others focused on older adults or specific athletic cohorts. Women, younger individuals across different training backgrounds, and elite athletes were not consistently represented across intervention categories, limiting the populations to which the findings can be directly applied. Intervention protocols ranged from single acute administrations to supplementation periods of up to 12 weeks, with studies conducted in the context of standardized exercise testing, habitual sports training, resistance training, or endurance events.

The main outcomes assessed included exercise performance and physiological efficiency, exercise- and training-related fatigue, muscle soreness and post-exercise recovery, skeletal-muscle strength and function, and training-related adaptations. Several trials also examined inflammatory, oxidative, immune, metabolic, mitochondrial, or skeletal-muscle molecular responses. The main characteristics and findings of the included studies are summarized in Table 1.

### 3.3. Risk of Bias in Included Studies

Risk of bias was assessed for one principal review-relevant outcome per independent trial. Of the 10 assessments, two were judged to be at low risk of bias [31,35], while eight raised some concerns. None was classified as high risk of bias. These judgments apply to the selected outcomes rather than to all outcomes reported within each trial.

The domains most consistently judged to be at low risk were deviations from intended interventions and outcome measurement, reflecting the use of placebo-controlled, blinded designs and standardized assessment procedures in most studies. Recurrent concerns arose from incomplete outcome data after randomization, insufficient reporting of sequence generation or allocation concealment, and potential selective reporting in exploratory trials. Small sample size was considered an issue of imprecision and generalizability rather than a RoB 2 domain and was therefore not used, by itself, to increase risk-of-bias judgments.

**Figure 2:**
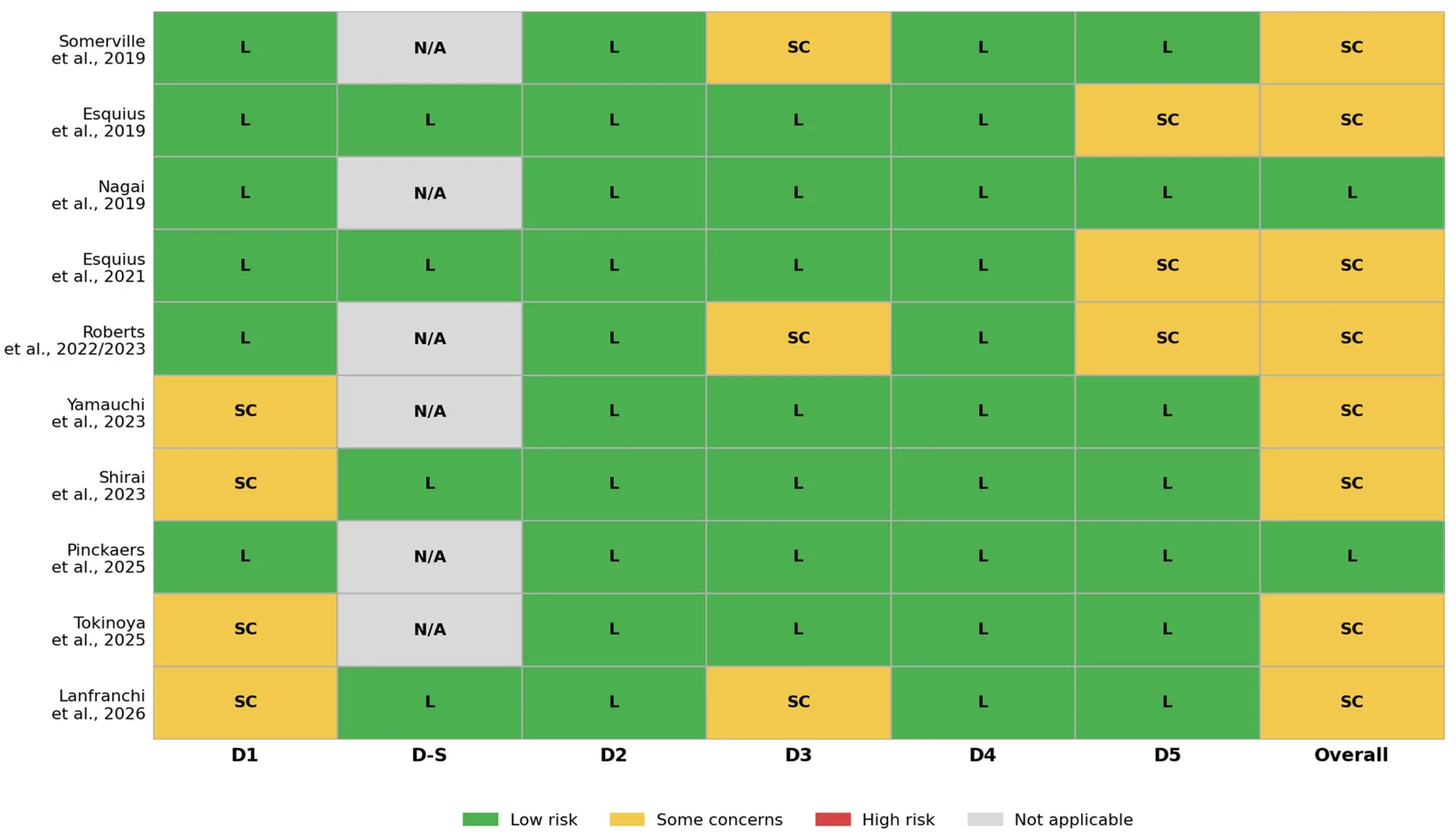
Traffic-light summary of RoB 2 judgments across included trials [13,26,29,30,31,32,33,34,35,36,37].

Most favorable findings for physiological efficiency, perceived fatigue, muscle soreness, and selected functional outcomes were reported in trials with some risk-of-bias concerns [26,30,32,33,34,36,37]. Among the two trials with low-risk assessments, improvements in selected muscle-mass measures were reported [31], whereas no improvements in leg strength or fatigue resistance were observed despite changes in skeletal-muscle metabolic activity [35]. These findings indicate that positive results were not exclusively confined to trials with methodological concerns; nevertheless, the predominance of such concerns reduces confidence in their reproducibility. Importantly, the outcome-specific nature of RoB 2 means that judgments should not automatically be extrapolated to other outcomes within the same trial. Domain-level assessments are presented in Table 2 and Figure 2.

The principal review-relevant outcome areas considered in the RoB 2 assessments were fatigue and wellbeing [29]; exercise performance and cardiorespiratory coordination [30]; skeletal-muscle mass and strength/function [31]; dendritic-cell responses during recovery [32]; aerobic performance, perceived exertion, and acute recovery [13,26]; grip strength and muscle mass [33]; perceived fatigue and muscle soreness [34]; leg strength and fatigue resistance [35]; muscle soreness and systemic fatigue at 24 h [36]; and sprint fatigue, power, and neuromuscular fatigability [37]. One overall RoB 2 judgment was reported per independent trial, based on a selected review-relevant result. These judgments should not be interpreted as separate risk-of-bias assessments for every outcome reported within each trial.

### 3.4. Exercise Performance and Physiological Efficiency

Exercise performance and physiological efficiency were assessed across different olive-derived interventions, with heterogeneous findings. In a small exploratory crossover trial (*n* = 7), higher values of the first principal component of cardiorespiratory coordination were observed following acute EVOO administration compared with palm oil (2.63 ± 0.51 vs. 2.30 ± 0.28; d = 0.80) and placebo (2.63 ± 0.51 vs. 2.38 ± 0.36; d = 0.57), although exercise duration and conventional performance measures did not differ between conditions [30]. Supplementation with the hydroxytyrosol-rich OliPhenolia^®^ preparation was associated with differences in oxygen-uptake kinetics (*p* = 0.005), running economy at the first lactate threshold (LT1; *p* = 0.015), and perceived exertion at the second lactate threshold (LT2; *p* = 0.003), without a statistically significant improvement in time to exhaustion [26]. No improvement in water-polo-specific performance was observed following maslinic-acid supplementation [34]. In the small sprint-interval subgroup of an acute olive-leaf-extract trial (*n* = 10 for performance analyses), the first-sprint fatigue index was 25.5 ± 29.1% lower than under placebo (*p* = 0.013), without significant differences in initial or repeated-sprint power [37]. Chronic olive-leaf-extract supplementation was not associated with significant improvements in leg strength or fatigue resistance [35]. Overall, these findings indicate isolated physiological or task-specific responses that require independent replication and do not establish consistent improvements in objective exercise performance.

### 3.5. Fatigue and Perceived Exertion

Fatigue-related findings varied according to the intervention and the outcome assessed. After adjustment for training load, the effect of olive-leaf extract on self-reported fatigue remained unclear [29]. OliPhenolia^®^ supplementation was associated with lower ratings of perceived exertion at the second lactate threshold compared with placebo (*p* = 0.003) [26]. In water-polo athletes, lower self-reported whole-body and muscle fatigue scores were observed during maslinic-acid supplementation (*p* < 0.05) [34]. Following marathon running, systemic fatigue did not differ significantly between groups immediately after exercise, whereas lower fatigue scores were reported in the maslinic-acid group at 24 h (*p* < 0.017) [36]. In contrast to these subjective measures, chronic olive-leaf-extract supplementation was not associated with improved objectively assessed muscle fatigue resistance [35]. Acute olive-leaf-extract supplementation was associated with a lower first-sprint fatigue index, an exercise-derived rather than self-reported measure, without corresponding improvements in sprint power or post-exercise knee-extensor fatigability [37]. Although perceived fatigue and exertion are relevant outcomes, they remain susceptible to expectancy and reporting effects, even in blinded trials. Consequently, these findings should be interpreted separately from objectively measured exercise performance and functional recovery.

### 3.6. Post-Exercise Recovery and Muscle Soreness

Muscle soreness and recovery-related outcomes were evaluated across different olive-derived interventions, with heterogeneous findings. Olive-leaf-extract supplementation showed no beneficial effect on muscle soreness, with the adjusted effect classified as possibly harmful after controlling for training load [29]. Supplementation with the hydroxytyrosol-rich OliPhenolia^®^ preparation was associated with a shorter respiratory recovery index (*p* = 0.02), although this physiological response does not necessarily indicate improved functional recovery [26]. Maslinic-acid supplementation was associated with reductions in whole-body and site-specific muscle soreness during repeated water-polo training (*p* < 0.05) [34]. In marathon runners, no significant between-group differences in muscle soreness were observed immediately after the race, whereas lower soreness was reported at several anatomical sites 24 h after the marathon in the maslinic-acid group (*p* < 0.017) [36]. However, circulating markers of muscle damage did not differ significantly between groups [36]. Overall, the reported differences in muscle soreness were based on self-reported measures and were limited to specific interventions, exercise contexts, and assessment time points. Although perceived soreness is relevant to athletes, these outcomes may be influenced by expectancy and reporting variability. Moreover, the absence of corresponding differences in muscle-damage biomarkers in the marathon trial [36] reinforces the need to distinguish perceived recovery from objectively demonstrated restoration of exercise capacity or athletic performance.

### 3.7. Skeletal-Muscle Function and Training Adaptation

Chronic maslinic-acid supplementation was associated with changes in selected measures of skeletal-muscle mass and strength, primarily in healthy older adults. Significant group × time interactions were reported for total skeletal-muscle mass and selected regional muscle-mass measures following maslinic-acid supplementation combined with resistance training [31]. Grip strength increased within the intervention group, although a significant between-group effect was not demonstrated [31]. In another randomized trial, grip strength was significantly higher than placebo following supplementation with both 30 and 60 mg/day of maslinic acid (*p* < 0.05), while trunk muscle mass was higher in the 60-mg/day group [33]. However, whole-body muscle mass and several physical-function outcomes remained unchanged [33]. In contrast, chronic olive-leaf-extract supplementation did not significantly improve leg strength or fatigue resistance compared with placebo, despite changes in skeletal-muscle metabolic activity [35]. Overall, these findings suggest that selected muscle-related outcomes may respond to specific olive-derived interventions, particularly maslinic acid in older adults, but the evidence remains limited and does not establish consistent improvements in skeletal-muscle function or training adaptation across interventions.

### 3.8. Exercise-Related Biochemical, Immune, and Mechanistic Responses

Several trials assessed immune, redox, inflammatory, and skeletal-muscle metabolic markers, with findings that require cautious interpretation. Olive-leaf-extract supplementation was not associated with a significant difference in upper respiratory illness incidence, although fewer sick days were reported in the intervention group (OR 0.72, 95% CI 0.56–0.93; *p* = 0.02) [29]. In an exploratory crossover trial involving only three participants, a 46% decrease in a mature myeloid dendritic-cell indicator was reported between 1 and 24 h after exercise under the EVOO condition (95% CI 30–62%; *p* = 0.05). However, this confidence interval describes a within-condition change rather than a demonstrated between-condition intervention effect, and the extremely small sample precludes reliable conclusions [32]. OliPhenolia^®^ supplementation was associated with differences in post-exercise reduced glutathione (GSH; *p* = 0.009) and superoxide dismutase (SOD) responses, without significant differences in malondialdehyde (MDA) or catalase (CAT) [13]. Selected inflammatory and oxidative-stress-related markers also differed between intervention conditions in the maslinic-acid trial involving water-polo athletes [34]. In a chronic olive-leaf-extract trial, skeletal-muscle fractional pyruvate dehydrogenase (PDH) activity was higher in the intervention group (49 ± 14% vs. 38 ± 10%; *p* = 0.016), whereas mitochondrial respiration and whole-body substrate metabolism were not significantly different [35]. No significant between-group differences in blood markers of muscle damage were observed following marathon running [36]. Finally, in an acute olive-leaf-extract trial involving small exercise-specific subgroups, differences in post-exercise PDH activity (*p* = 0.0059) and selected transcriptomic responses were reported after moderate-intensity continuous exercise [37]. These exploratory findings do not establish clinically meaningful physiological benefits or enhanced exercise performance.

Across the included trials, biochemical, immune, inflammatory, and metabolic responses were heterogeneous and specific to the intervention and outcome assessed. Differences in selected oxidative and inflammatory markers were reported following OliPhenolia^®^ and maslinic-acid supplementation [13,34], while olive-leaf-extract administration was associated with changes in skeletal-muscle pyruvate dehydrogenase activity under specific experimental conditions [35,37]. However, these biochemical findings were not consistently accompanied by improvements in objective exercise performance, muscle strength, fatigue resistance, or functional recovery. Similarly, lower self-reported fatigue and muscle soreness scores in selected maslinic-acid trials [34,36] should not be considered equivalent to demonstrated improvements in physical performance. Importantly, the direction of a biochemical or inflammatory response does not, by itself, establish whether the change is beneficial, detrimental, or physiologically adaptive. Therefore, perceptual outcomes, mechanistic biomarkers, and objective functional measures were interpreted as distinct evidence domains, and no generalized performance-enhancing effect can be inferred from the available findings.

### 3.9. Certainty of Evidence

The GRADE assessment indicated predominantly low or very low certainty across the main exercise-related outcomes (Table 3). Evidence for leg strength and fatigue resistance following chronic olive-leaf-extract supplementation was rated as low certainty, with no statistically significant improvements observed in the contributing trial. Evidence for selected muscle-mass outcomes following maslinic-acid supplementation was also rated as low certainty. Most other intervention–outcome comparisons were rated as very low certainty, reflecting methodological concerns, imprecision, and limited replication across independent trials. These findings indicate substantial uncertainty regarding the reproducibility and practical relevance of the reported effects and do not establish a general ergogenic benefit of olive-derived interventions.

## 4. Discussion

This systematic review synthesized evidence from 10 independent randomized controlled trials of olive-derived nutritional interventions across exercise performance, fatigue, recovery, skeletal-muscle outcomes, and exercise-related physiological responses. The principal finding is that these interventions have not consistently improved conventional measures of exercise performance. Favorable responses were observed for selected physiological, perceptual, muscular, and biochemical outcomes, but their direction and magnitude varied across formulations, doses, populations, and exercise settings. These findings require an intervention- and outcome-specific interpretation rather than suggesting a common ergogenic effect of olive-derived products. In particular, changes in soreness, perceived exertion, or biochemical markers should be distinguished from outcomes that demonstrate improved sport-specific performance or sustained training capacity.

The distinction between physiological responses and objective performance is particularly important. Acute extra virgin olive oil (EVOO) administration improved cardiorespiratory coordination without increasing exercise duration [30]. A hydroxytyrosol-rich olive-fruit-water preparation (OliPhenolia^®^) improved oxygen-uptake kinetics and running economy and reduced perceived exertion, but did not significantly improve time to exhaustion compared with placebo [26]. Acute olive-leaf extract reduced the first-sprint fatigue index without improving sprint power [37], while maslinic acid did not improve water-polo-specific performance [34]. Thus, changes in intermediate physiological measures cannot be assumed to reflect greater exercise capacity. The lack of consistent effects on exercise duration, time to exhaustion, and sprint power limits the interpretation of these interventions as performance-enhancing strategies. Although broader evidence on polyphenol supplementation suggests possible improvements in selected endurance outcomes, differences between preparations and protocols preclude direct extrapolation to the interventions examined here [38].

Perceived fatigue and muscle soreness showed favorable but context-dependent responses in selected maslinic-acid trials [34,36]. Although these outcomes are relevant to athletes, they rely on self-report and may be influenced by expectancy, contextual factors, and reporting effects, even in double-blind, placebo-controlled studies. Blinding reduces the potential for such influences but does not necessarily eliminate them. This possibility does not establish that the observed differences were caused by placebo effects; rather, it reinforces the need for cautious interpretation, particularly when subjective improvements are not accompanied by objective functional changes. For example, lower perceived soreness and fatigue at 24 h after marathon running were not accompanied by significant between-group differences in circulating muscle-damage markers [36]. Broader evidence concerning polyphenol-rich interventions also suggests that perceived soreness may change without corresponding improvements in performance or muscle-damage biomarkers [39]. However, this evidence cannot be directly extrapolated to maslinic acid, which is a pentacyclic triterpene rather than a polyphenol [10]. Thus, perceived improvements should be considered alongside objective measures of exercise capacity, recovery, and training adaptation rather than treated as equivalent evidence of ergogenic efficacy.

Findings for olive-leaf extracts further illustrate why different measures of fatigue should not be treated as interchangeable. During habitual sports training, effects on fatigue were unclear and the adjusted effect on soreness was classified as possibly harmful [29]. Acute supplementation reduced a sprint-specific fatigue index [37], whereas chronic supplementation in older men did not improve leg strength or fatigue resistance [35]. Differences in intervention duration, population, exercise stimulus, and outcome definition prevent these results from being considered direct replications or necessarily contradictory findings. Perceived fatigue during a competitive season, fatigue resistance during standardized muscle testing, and a fatigue index calculated from repeated sprints represent different constructs. Accordingly, a favorable result in one setting offers limited predictive information about another.

Skeletal-muscle outcomes were investigated mainly with maslinic acid in older adults. One trial reported favorable group-by-time interactions for total and regional muscle mass during resistance training, although the improvement in grip strength was observed within the intervention group rather than as a significant between-group difference [31]. Another reported greater grip strength at 30 and 60 mg/day and higher trunk muscle mass at 60 mg/day, but no change in several other functional outcomes [33]. These findings do not establish a consistent effect on strength, whole-body muscle mass, or training adaptation, particularly in younger or highly trained individuals. The selected gains in regional muscle mass should also be interpreted alongside outcomes that did not differ from placebo, and the interaction between supplementation and concurrent physical training cannot be assumed to apply to other training programs. Similarly, broader evidence on chronic polyphenol supplementation does not provide a basis for assuming additional resistance-training adaptations [40]; moreover, polyphenols and maslinic acid are chemically distinct.

Biochemical and molecular findings were also selective. OliPhenolia^®^ altered post-exercise glutathione and superoxide dismutase responses without consistent effects on malondialdehyde or catalase [13]. Maslinic acid modified selected inflammatory and oxidative markers in water-polo athletes [34], while an exploratory EVOO crossover study involving only three participants reported a change in dendritic-cell responses [32]. Olive-leaf extract increased skeletal-muscle pyruvate dehydrogenase activity under certain conditions, without corresponding changes in whole-body metabolism, strength, or fatigue resistance [35,37]. Such biomarker changes cannot be equated with improved recovery or training adaptation. Importantly, the direction of a biomarker change is not intrinsically favorable: the biological relevance of an altered inflammatory or oxidative response depends on its timing, magnitude, and relationship with subsequent functional outcomes. Exercise-related redox signaling is involved in physiological adaptation, and a change in oxidative or inflammatory markers is not inherently beneficial or detrimental [1,2,5,41].

The nutritional matrix is therefore essential to causal interpretation. EVOO is a lipid-rich whole-food intervention containing oleic acid and numerous minor constituents, including phenolics, tocopherols, and phytosterols [7,8,9]. Its observed effects [30,32] cannot be attributed specifically to hydroxytyrosol or any single polyphenol because the contribution of the complete matrix was not isolated. OliPhenolia^®^ is also a multicomponent formulation; the results from its trial apply to the tested preparation rather than to purified hydroxytyrosol [13,26]. Olive-leaf extracts, typically enriched in oleuropein, and maslinic-acid preparations differ substantially in chemical composition and plausible mechanisms [10,11]. Findings for one preparation therefore cannot be generalized to other products merely because they share an olive origin. Variation in olive cultivar, processing, extraction, standardization, and co-ingredients may further alter exposure to active constituents. Without direct component-isolation or head-to-head trials, neither a common mechanism nor equivalence between whole-food products, extracts, and isolated compounds can be established.

This review extends previous work without implying comparability between chemically distinct interventions. Evidence concerning Mediterranean dietary patterns cannot isolate the contribution of olive-derived products [19,20], while earlier olive-focused reviews emphasized EVOO in athletes or olive polyphenols and inflammatory responses [21,22]. By restricting inclusion to randomized human trials and distinguishing performance, perceptual, functional, and biochemical endpoints, the present review identifies both selected signals of potential benefit and the substantial gaps between such signals and demonstrated ergogenic effects. This distinction is especially important given that trials of different olive-derived formulations cannot be combined as though they evaluated a single active intervention.

Confidence in the observed benefits is limited. Of the 10 outcome-specific RoB 2 assessments, two were rated as low risk and eight as having some concerns. Most favorable findings arose from trials with methodological concerns, including incomplete reporting of randomization procedures, missing outcome data, or uncertainty about the selection of reported results. Small sample sizes further increase imprecision, although sample size is not itself a RoB 2 domain [27]. Among the two low-risk assessments, one trial reported favorable changes in selected muscle-mass outcomes, whereas the other found no improvements in strength or fatigue resistance [31,35]. Importantly, because RoB 2 was applied to only one principal review-relevant result per trial, these judgments cannot be generalized to other endpoints. Several trials assessed multiple physiological, perceptual, functional, and biochemical outcomes, sometimes at different time points, without consistently available prespecified outcome definitions or analysis plans. Consequently, selective reporting of statistically significant findings, potential nonreporting of nonsignificant outcomes, and chance findings arising from multiple comparisons cannot be excluded, particularly in small exploratory trials. Although the present synthesis considered reported null findings alongside favorable results, this approach cannot eliminate potential selective outcome reporting in the original studies.

Independent replication also remains limited. The two publications reporting outcomes following OliPhenolia^®^ supplementation originated from the same randomized trial and therefore do not provide independent confirmation [13,26]. For maslinic acid, selected muscle-related effects were reported in two independent trials involving older adults [31,33], whereas reductions in perceived fatigue or muscle soreness were observed in separate studies involving water-polo athletes and marathon runners [34,36]. However, differences in populations, exercise protocols, supplementation strategies, and outcome assessments limit their comparability and prevent these findings from being considered direct replications. Together with the methodological concerns and the possibility of selective outcome reporting, these limitations reduce confidence in the reproducibility of isolated positive findings. Furthermore, the small number of comparable trials prevents a reliable assessment of publication bias. Consequently, the available positive findings should be considered preliminary and hypothesis-generating rather than conclusive evidence of efficacy.

The magnitude and duration of an effect are also critical to practical relevance. Reduced soreness and fatigue 24 h after a marathon were not accompanied by demonstrated improvements in subsequent training capacity [36]. A 25.5 ± 29.1% reduction in the first-sprint fatigue index did not translate into higher sprint power [37], while greater fractional pyruvate dehydrogenase activity (49 ± 14% vs. 38 ± 10%) was not accompanied by improved strength, fatigue resistance, or whole-body metabolism [35]. Similarly, favorable aerobic physiological responses with OliPhenolia^®^ did not result in a significant improvement in time to exhaustion [26]. None of these findings alone establishes a meaningful advantage for competitive performance, restoration of exercise capacity, or long-term adaptation. Moreover, clinically or practically important differences were generally not established in advance, and the available reporting does not consistently support an appraisal of precision against such thresholds. An observed percentage change or statistically significant test result is therefore insufficient to determine whether an effect is useful in practice.

The interpretation and generalizability of these findings are limited by substantial heterogeneity in product composition, dose, supplementation timing, exercise protocols, study populations, and outcome definitions, which also precluded meaningful quantitative pooling. Another important limitation is that habitual consumption of olive oil and other polyphenol-rich foods was not consistently assessed, reported, or controlled across the included trials. Differences in baseline dietary exposure may have modified individual responses to supplementation, increased variability, and complicated the interpretation of intervention-related effects, particularly in small randomized trials. Although randomization can reduce confounding, imbalances in background dietary intake cannot be excluded when such information is unavailable. Furthermore, several trials included exclusively male participants or focused on healthy older adults or narrowly defined athletic populations.

Consequently, the generalizability of these findings to women, younger individuals with different training characteristics, and elite athletes remains uncertain.

Initial title and abstract screening, full-text assessment, and data extraction were performed by a single reviewer. However, a second reviewer independently verified full-text eligibility decisions, checked the extracted data, and corroborated the RoB 2 assessments. The absence of independent duplicate title and abstract screening remains a methodological limitation. The review was not prospectively registered and did not have a published protocol. Strengths include restriction to randomized trials, searches of multiple databases and trial registries, use of parallel- and crossover-specific RoB 2 assessments, and avoidance of double-counting the two reports from one trial. Nevertheless, the evidence base remains too small and heterogeneous to estimate a shared intervention effect.

Future trials should be independently replicated, adequately powered, and prospectively registered, with prespecified primary outcomes and analysis plans. They should quantify the composition and dose of the tested preparation, document background dietary intake and adherence, and use objective, sport-relevant performance measures alongside validated fatigue and recovery outcomes. Longer follow-up is needed to establish whether short-term biochemical or perceptual responses translate into durable functional improvements or training adaptations. Greater representation of women, competitive athletes, and diverse age groups, together with transparent reporting of effect sizes, confidence intervals, adverse events, and tolerability, would strengthen the clinical and practical applicability of the evidence. Designs should also distinguish short-term physiological changes from improved recovery of performance after exercise, and should clearly report relevant null findings alongside favorable results.

## 5. Conclusions

The available randomized controlled evidence indicates that olive-derived nutritional interventions should be interpreted as chemically distinct preparations rather than as a single intervention category with shared physiological or ergogenic effects. Maslinic-acid supplementation was associated with lower perceived fatigue and muscle soreness in specific exercise settings and with selected muscle-related outcomes in older adults. In contrast, extra virgin olive oil, hydroxytyrosol-rich olive-fruit-water preparations, and oleuropein-rich olive-leaf extracts showed heterogeneous responses across physiological, metabolic, and fatigue-related outcomes. These findings are specific to the formulations, doses, populations, and exercise protocols investigated and cannot be generalized across olive-derived products. Moreover, selected perceptual or biochemical responses do not establish consistent improvements in objective exercise performance, functional recovery, or training adaptation.

Accordingly, olive-derived foods, extracts, and isolated compounds should not currently be regarded as a broadly ergogenic nutritional strategy. Future research should prioritize adequately powered, prospectively registered randomized controlled trials using standardized, chemically characterized formulations with clearly quantified bioactive composition, dosage, supplementation duration, and timing. Studies should include prespecified, objective, sport-relevant performance outcomes, validated measures of fatigue and recovery, and sufficient follow-up to assess the persistence of effects and potential training adaptations. Adherence, adverse events, and tolerability should also be systematically monitored and transparently reported. These methodological improvements are essential before evidence-based recommendations can be established for individual olive-derived interventions.

## Figures and Tables

**Table 1:** Characteristics and main findings of randomized controlled trials evaluating olive-derived nutritional interventions in exercise performance, fatigue, recovery, and skeletal-muscle function.

Study	Design and Participants	Olive-Derived Intervention	Comparator and Duration	Exercise/Training Context	Main Outcomes	Main Findings
Somerville et al., 2019 [29]	Randomized controlled trial; 32 high-school athletes competing for elite school teams	Olive-leaf extract equivalent to 20 g olive leaf, providing 100 mg/day oleuropein	Placebo; 9 weeks during the competitive season	Habitual sport training and competition; training load recorded twice weekly	Wellbeing (soreness, stress, sleep quality, fatigue, satiety), training load, upper respiratory illness	No significant reduction in upper respiratory illness incidence. Fewer sick days were reported (OR 0.72, 95% CI 0.56–0.93; *p* = 0.02). After adjustment for training load, the effect on soreness was classified as possibly harmful, while effects on fatigue remained unclear.
Esquius et al., 2019 [30]	Randomized, double-blind, crossover controlled trial; *n* = 7 physically active men	25 mL EVOO, administered 1 h before exercise	Palm oil and placebo; acute intervention with 7-day washout between conditions	Progressive maximal walking test	Cardiorespiratory coordination, physiological variables, exercise duration/performance	In this exploratory crossover trial (*n* = 7), higher cardiorespiratory coordination values were observed under EVOO than palm oil (d = 0.80) or placebo (d = 0.57). Exercise duration and conventional performance outcomes did not differ significantly.
Nagai et al., 2019 [31]	Parallel, double-blind, randomized placebo-controlled trial; *n* = 36 community-dwelling older adults completing the study	Olive-fruit-derived maslinic acid, 60 mg/day	Placebo; 12 weeks	Moderate resistance-training program	Skeletal and segmental muscle mass, grip strength, leg strength, walking speed, mobility, knee pain	Favorable group-by-time interactions were observed for selected skeletal-muscle mass measures. Grip strength increased within the intervention group, without a demonstrated significant between-group effect.
Esquius et al., 2021 [32]	Randomized, double-blind, crossover controlled pilot trial; *n* = 3 physically active men	25 mL EVOO	Placebo; acute intervention, conditions separated by 1 week	Strenuous exercise protocol	Dendritic-cell maturation, inflammatory/immune response, metabolic variables, 24-h recovery	In this exploratory trial (*n* = 3), mature myeloid dendritic-cell levels decreased by approximately 46% from the post-exercise peak to 24 h within the EVOO condition (95% CI 30–62%). A between-condition immune benefit was not established.
Roberts et al., 2022; Roberts et al., 2023 † [13,26]	Randomized, double-blind, placebo-controlled trial; *n* = 29 recreationally active adults	Olive-fruit-water phytocomplex (OliPhenolia^®^), hydroxytyrosol-rich; 2 × 28 mL/day	Matched placebo; 16 days	Standardized aerobic exercise (~75% VO_2_max), followed by submaximal and maximal exercise testing.	Oxidative-stress markers, running economy, VO_2_ kinetics, RPE, time to exhaustion, acute recovery	Differences were reported in post-exercise GSH (*p* = 0.009), oxygen-uptake kinetics (*p* = 0.005), running economy (*p* = 0.015), perceived exertion (*p* = 0.003), and a respiratory recovery index (*p* = 0.02). Time to exhaustion did not differ significantly from placebo.
Yamauchi et al., 2023 [33]	Randomized, double-blind, placebo-controlled trial; *n* = 69 healthy Japanese older adults	Olive-derived maslinic acid, 30 or 60 mg/day	Placebo; 12 weeks	Physical-exercise program during supplementation	Grip strength, trunk muscle mass, physical function, quality of life	Grip strength was higher than placebo at both 30 and 60 mg/day, and trunk muscle mass was higher at 60 mg/day (*p* < 0.05). Other physical-function outcomes showed no consistent differences.
Shirai et al., 2023 [34]	Randomized, double-blind, crossover, placebo-controlled trial; *n* = 12 young male water-polo athletes	Olive-fruit extract providing 60 mg/day maslinic acid	Placebo; 7-day training periods	Daily water-polo training	Perceived fatigue, muscle soreness, inflammatory and oxidative-stress-related responses	Lower self-reported fatigue and muscle soreness scores were observed during maslinic-acid supplementation (*p* < 0.05), alongside selected inflammatory and oxidative-stress-related differences. Sport-specific performance did not improve.
Pinckaers et al., 2025 [35]	Randomized, double-blind, placebo-controlled parallel trial; *n* = 40 healthy older men (60 ± 5 years)	250 mg/day olive-leaf extract providing 100 mg/day oleuropein	Placebo; 36 days	Standardized assessments of leg strength, fatigue resistance, skeletal-muscle metabolism and whole-body metabolism	Pyruvate dehydrogenase (PDH) activity, mitochondrial respiration, strength, fatigue resistance, whole-body metabolism	Skeletal-muscle fractional PDH activity was higher under olive-leaf extract than placebo (49 ± 14% vs. 38 ± 10%; *p* = 0.016). Muscle strength, fatigue resistance, mitochondrial respiration, and whole-body metabolism did not differ significantly.
Tokinoya et al., 2025 [36]	Randomized, double-blind, placebo-controlled trial; *n* = 27 healthy amateur marathon runners (19 men, 8 women); 22–26 analyzed depending on outcome	Olive-derived maslinic acid, 60 mg/day	Placebo; started 5 days before the marathon and continued through the race period	Full marathon	Muscle soreness, systemic fatigue, blood markers of muscle damage/physiological stress	Lower perceived soreness and systemic fatigue were observed 24 h after marathon running (*p* < 0.017), but not immediately after exercise. Blood markers of muscle damage did not differ significantly between groups.
Lanfranchi et al., 2026 [37]	Randomized, double-blind, crossover trial; 22 healthy physically active men allocated to MICE (*n* = 11) or SIE (*n* = 11; performance analyses *n* = 10 for some outcomes)	250 mg olive-leaf extract containing 100 mg oleuropein, acute administration	Placebo; crossover with 10–18-day washout	MICE: 1 h cycling at 50% maximal aerobic power; SIE: 6 × 30-s all-out sprints with 4-min recovery	Power output, fatigue index, heart rate, neuromuscular fatigue, skeletal-muscle PDH activity, transcriptomic and mitochondrial responses	In a small sprint-analysis subgroup, the first-sprint fatigue index was 25.5 ± 29.1% lower under olive-leaf extract (*p* = 0.013), without improvements in sprint power or knee-extensor fatigability. Selected PDH and molecular responses differed after moderate-intensity exercise.

Note. † Roberts et al. [13] and Roberts et al. [26] reported complementary outcomes from the same intervention trial and were therefore treated as one independent study. Abbreviations: EVOO, extra virgin olive oil; OLE, olive-leaf extract; URI, upper respiratory illness; OR, odds ratio; CI, confidence interval; PC1, first principal component; VO_2_max, maximal oxygen uptake; LT1, first lactate threshold; LT2, second lactate threshold; RPE, rating of perceived exertion; GSH, reduced glutathione; SOD, superoxide dismutase; MDA, malondialdehyde; CAT, catalase; PDH, pyruvate dehydrogenase; MICE, moderate-intensity continuous exercise; SIE, sprint-interval exercise.

**Table 2:** Risk-of-bias assessment for the selected principal review-relevant result from each included randomized controlled trial using Cochrane RoB 2.

Study	D1	D-S	D2	D3	D4	D5	Overall
Somerville et al. [29]	Low	N/A	Low	Some concerns	Low	Low	Some concerns
Esquius et al. [30]	Low	Low	Low	Low	Low	Some concerns	Some concerns
Nagai et al. [31]	Low	N/A	Low	Low	Low	Low	Low
Esquius et al. [32]	Low	Low	Low	Low	Low	Some concerns	Some concerns
Roberts et al. [13,26]	Low	N/A	Low	Some concerns	Low	Some concerns	Some concerns
Yamauchi et al. [33]	Some concerns	N/A	Low	Low	Low	Low	Some concerns
Shirai et al. [34]	Some concerns	Low	Low	Low	Low	Low	Some concerns
Pinckaers et al. [35]	Low	N/A	Low	Low	Low	Low	Low
Tokinoya et al. [36]	Some concerns	N/A	Low	Low	Low	Low	Some concerns
Lanfranchi et al. [37]	Some concerns	Low	Low	Some concerns	Low	Low	Some concerns

Note. Background colors indicate risk-of-bias judgments: green, low risk; yellow, some concerns; gray, not applicable. Abbreviations: D1, bias arising from the randomization process; D-S, bias arising from period and carryover effects in crossover trials; D2, bias due to deviations from intended interventions; D3, bias due to missing outcome data; D4, bias in measurement of the outcome; D5, bias in selection of the reported result; N/A, not applicable.

**Table 3:** GRADE assessment of the certainty of evidence for the main exercise-related outcomes.

Intervention/Outcome	Contributing Studies	Summary of Findings	GRADE Certainty	Main Reasons for Downgrading
EVOO—Exercise performance	[30]	No statistically significant difference in exercise duration or conventional performance outcomes.	⊕◯◯◯ Very low	Methodological concerns; very serious imprecision (*n* = 7).
EVOO—Cardiorespiratory coordination	[30]	Higher coordination values during moderate-intensity exercise; functional relevance uncertain.	⊕◯◯◯ Very low	Methodological concerns; very serious imprecision (*n* = 7).
Olive-fruit-water—Time to exhaustion	[26]	No statistically significant between-group improvement.	⊕◯◯◯ Very low	Methodological concerns; very serious imprecision; single small trial.
Olive-fruit-water—Running economy	[26]	Differences in running economy at LT1, without demonstrated improvement in time to exhaustion.	⊕◯◯◯ Very low	Methodological concerns; very serious imprecision; no independent replication.
Olive-fruit-water—Perceived exertion	[26]	Lower self-reported exertion at LT2; performance relevance uncertain.	⊕◯◯◯ Very low	Methodological concerns; very serious imprecision; subjective outcome.
Olive-fruit-water—Respiratory recovery	[26]	Shorter respiratory recovery index, without demonstrated improvement in functional recovery.	⊕◯◯◯ Very low	Methodological concerns; very serious imprecision; indirect relevance to functional recovery.
Olive-leaf extract—Perceived fatigue	[29]	Effect on self-reported fatigue remained unclear after adjustment for training load.	⊕◯◯◯ Very low	Methodological concerns; very serious imprecision; subjective outcome.
Olive-leaf extract—Muscle soreness	[29]	Adjusted effect on soreness was classified as possibly harmful.	⊕◯◯◯ Very low	Methodological concerns; very serious imprecision; subjective outcome.
Olive-leaf extract—Leg strength	[35]	No statistically significant between-group improvement.	⊕⊕◯◯ Low	Very serious imprecision; one trial with a limited sample.
Olive-leaf extract—Fatigue resistance	[35]	No statistically significant between-group improvement.	⊕⊕◯◯ Low	Very serious imprecision; one trial with a limited sample.
Olive-leaf extract—First-sprint fatigue index	[37]	Lower first-sprint fatigue index in a small subgroup; no corresponding improvement in sprint power.	⊕◯◯◯ Very low	Methodological concerns; very serious imprecision; exploratory subgroup.
Olive-leaf extract—Repeated-sprint power	[37]	No consistent between-condition improvement.	⊕◯◯◯ Very low	Methodological concerns; very serious imprecision; small subgroup.
Maslinic acid—Grip strength	[31,33]	Inconsistent evidence: a within-group increase in one trial and a between-group difference in another.	⊕◯◯◯ Very low	Methodological concerns; imprecision; inconsistency in the evidence of between-group effects.
Maslinic acid—Muscle mass	[31,33]	Differences in selected muscle-mass measures in older adults; effects were not consistent across all measures.	⊕⊕◯◯ Low	Methodological concerns; imprecision; limited comparability of muscle-mass outcomes.
Maslinic acid—Perceived fatigue	[34,36]	Lower self-reported fatigue in specific exercise settings or assessment periods.	⊕◯◯◯ Very low	Methodological concerns; very serious imprecision; heterogeneous exercise settings.
Maslinic acid—Muscle soreness	[34,36]	Lower self-reported soreness in selected exercise contexts and at specific time points.	⊕◯◯◯ Very low	Methodological concerns; very serious imprecision; heterogeneous assessment conditions.
Maslinic acid—Water-polo-specific performance	[34]	No statistically significant improvement in eggbeater-kick performance.	⊕◯◯◯ Very low	Methodological concerns; very serious imprecision; small single trial.

Note. GRADE, Grading of Recommendations Assessment, Development and Evaluation; EVOO, extra virgin olive oil; LT1, first lactate threshold; LT2, second lactate threshold. The symbols ⊕ and ◯ represent certainty ratings according to GRADE: ⊕⊕⊕⊕, high; ⊕⊕⊕◯, moderate; ⊕⊕◯◯, low; and ⊕◯◯◯, very low. Certainty ratings refer to specific intervention–outcome comparisons and should not be generalized across chemically distinct olive-derived products. Evidence from randomized trials was initially considered high certainty and evaluated for risk of bias, inconsistency, indirectness, imprecision, and publication bias. Inconsistency was not automatically downgraded when only one trial contributed to an outcome. Lack of statistical significance was not interpreted as evidence of no effect, particularly when confidence intervals were unavailable or did not exclude clinically meaningful differences. Publication bias could not be formally assessed because of the small number of comparable studies.

## Data Availability

No new data were created or generated in this study. All data supporting the findings of this systematic review were derived from the published studies included in the review and are reported within the article and its Supplementary Materials.

## Supplementary Materials

The following supporting information can be downloaded at: https://www.mdpi.com/article/10.3390/nu18213699/s1, Table S1: Complete search strategies for all databases and trial registries, final search dates, and numbers of records retrieved; Table S2: Retrospective summary of review methods and synthesis decisions.

## Abbreviations

The following abbreviations are used in this manuscript:

EVOO	Extra virgin olive oil
GSH	Reduced glutathione
LT1	First lactate threshold
LT2	Second lactate threshold/lactate turn point
MDA	Malondialdehyde
MICE	Moderate-intensity continuous exercise
OLE	Olive-leaf extract
PC1	First principal component
PDH	Pyruvate dehydrogenase
RPE	Rating of perceived exertion
SIE	Sprint interval exercise
SOD	Superoxide dismutase
URI	Upper respiratory illness
VO_2_	Oxygen uptake
CAT	Catalase
