# Supplementary material for: Olive-Derived Nutritional Interventions in Exercise: Effects on Performance, Fatigue, Recovery, and Muscle Adaptation—A Systematic Review
Source: Nutrients. 2026 Nov 7;18(21):3699. doi: 10.3390/nu18213699 (PMC13765110; doi:10.3390/nu18213699)
Supplement: Supplementary file 1 [file nutrients-18-03699-s001.zip › Supplementary Table S1.pdf]

Supplementary Table S1. Complete Search Strategies Used in the Systematic Review

Information source	Records retrieved
MEDLINE via PubMed	238
Scopus	326
Web of Science Core Collection	287
SPORTDiscus via EBSCOhost	128
CENTRAL	147
Total bibliographic databases	1,126
ClinicalTrials.gov	13
WHO ICTRP	8
Total trial registries	21

Strategy 1. MEDLINE via PubMed

Date of final search: 30 September 2026.

("Olive Oil"[Mesh] OR "olive oil"[tiab] OR "olive oils"[tiab] OR
"extra virgin olive oil"[tiab] OR "extra-virgin olive oil"[tiab] OR
EVOO[tiab] OR "Olea europaea"[tiab] OR "olive fruit"[tiab] OR
"olive fruit extract"[tiab] OR "olive fruit extracts"[tiab] OR
"olive fruit water"[tiab] OR "olive leaf"[tiab] OR "olive leaves"[tiab] OR
"olive leaf extract"[tiab] OR "olive leaf extracts"[tiab] OR
"olive-derived"[tiab] OR "olive derived"[tiab] OR
"olive polyphenol"[tiab] OR "olive polyphenols"[tiab] OR
"olive phenolic"[tiab] OR "olive phenolics"[tiab] OR
hydroxytyrosol[tiab] OR oleuropein[tiab] OR oleocanthal[tiab] OR
oleacein[tiab] OR "maslinic acid"[tiab] OR OliPhenolia[tiab])
AND
("Exercise"[Mesh] OR "Sports"[Mesh] OR "Athletes"[Mesh] OR
exercis*[tiab] OR sport*[tiab] OR athlete*[tiab] OR train*[tiab] OR

"physical activity"[tiab] OR "physical performance"[tiab] OR

"exercise performance"[tiab] OR endurance[tiab] OR strength[tiab] OR

fatigue[tiab] OR recovery[tiab] OR "muscle soreness"[tiab] OR

"muscle damage"[tiab] OR "muscle function"[tiab] OR

"skeletal muscle"[tiab] OR "muscle mass"[tiab] OR

"fatigue resistance"[tiab] OR "running economy"[tiab] OR

"time to exhaustion"[tiab] OR "oxygen uptake"[tiab] OR VO2[tiab] OR

"perceived exertion"[tiab] OR "training adaptation"[tiab])

Strategy 2. Scopus

Date of final search: 30 September 2026.

TITLE-ABS-KEY(

"olive oil" OR "olive oils" OR "extra virgin olive oil" OR

"extra-virgin olive oil" OR EVOO OR "Olea europaea" OR

"olive fruit" OR "olive fruit extract" OR "olive fruit extracts" OR

"olive fruit water" OR "olive leaf" OR "olive leaves" OR

"olive leaf extract" OR "olive leaf extracts" OR

"olive-derived" OR "olive derived" OR

"olive polyphenol" OR "olive polyphenols" OR

"olive phenolic" OR "olive phenolics" OR

hydroxytyrosol OR oleuropein OR oleocanthal OR oleacein OR

"maslinic acid" OR OliPhenolia

)

AND

TITLE-ABS-KEY(

exercis* OR sport* OR athlete* OR train* OR

"physical activity" OR "physical performance" OR

"exercise performance" OR endurance OR strength OR fatigue OR

recovery OR "muscle soreness" OR "muscle damage" OR

"muscle function" OR "skeletal muscle" OR "muscle mass" OR

"fatigue resistance" OR "running economy" OR

"time to exhaustion" OR "oxygen uptake" OR VO2 OR

"perceived exertion" OR "training adaptation"

)

Strategy 3. Web of Science Core Collection

Date of final search: 30 September 2026.

TS=(

"olive oil" OR "olive oils" OR "extra virgin olive oil" OR

"extra-virgin olive oil" OR EVOO OR "Olea europaea" OR

"olive fruit" OR "olive fruit extract" OR "olive fruit extracts" OR

"olive fruit water" OR "olive leaf" OR "olive leaves" OR

"olive leaf extract" OR "olive leaf extracts" OR

"olive-derived" OR "olive derived" OR

"olive polyphenol" OR "olive polyphenols" OR

"olive phenolic" OR "olive phenolics" OR

hydroxytyrosol OR oleuropein OR oleocanthal OR oleacein OR

"maslinic acid" OR OliPhenolia

)

AND

TS=(

exercis* OR sport* OR athlete* OR train* OR

"physical activity" OR "physical performance" OR

"exercise performance" OR endurance OR strength OR fatigue OR

recovery OR "muscle soreness" OR "muscle damage" OR

"muscle function" OR "skeletal muscle" OR "muscle mass" OR

"fatigue resistance" OR "running economy" OR

"time to exhaustion" OR "oxygen uptake" OR VO2 OR

"perceived exertion" OR "training adaptation"

)

Strategy 4. SPORTDiscus via EBSCOhost

Date of final search: 30 September 2026.

TX (

"olive oil" OR "olive oils" OR "extra virgin olive oil" OR

"extra-virgin olive oil" OR EVOO OR "Olea europaea" OR

"olive fruit" OR "olive fruit extract" OR "olive fruit extracts" OR

"olive fruit water" OR "olive leaf" OR "olive leaves" OR

"olive leaf extract" OR "olive leaf extracts" OR

"olive-derived" OR "olive derived" OR

"olive polyphenol" OR "olive polyphenols" OR

"olive phenolic" OR "olive phenolics" OR

hydroxytyrosol OR oleuropein OR oleocanthal OR oleacein OR

"maslinic acid" OR OliPhenolia

)

AND

TX (

exercis* OR sport* OR athlete* OR train* OR

"physical activity" OR "physical performance" OR

"exercise performance" OR endurance OR strength OR fatigue OR

recovery OR "muscle soreness" OR "muscle damage" OR

"muscle function" OR "skeletal muscle" OR "muscle mass" OR

"fatigue resistance" OR "running economy" OR

"time to exhaustion" OR "oxygen uptake" OR VO2 OR

"perceived exertion" OR "training adaptation"

)

Strategy 5. Cochrane CENTRAL

Date of final search: 30 September 2026.

#1 [mh "Olive Oil"]

#2 ("olive oil" OR "olive oils" OR "extra virgin olive oil" OR

"extra-virgin olive oil" OR EVOO OR "Olea europaea" OR

"olive fruit" OR "olive fruit extract" OR "olive fruit extracts" OR

"olive fruit water" OR "olive leaf" OR "olive leaves" OR

"olive leaf extract" OR "olive leaf extracts" OR

"olive-derived" OR "olive derived" OR

"olive polyphenol" OR "olive polyphenols" OR

"olive phenolic" OR "olive phenolics" OR

hydroxytyrosol OR oleuropein OR oleocanthal OR oleacein OR

"maslinic acid" OR OliPhenolia):ti,ab,kw

#3 #1 OR #2

#4 [mh Exercise]

#5 [mh Sports]

#6 [mh Athletes]

#7 (exercis* OR sport* OR athlete* OR train* OR

"physical activity" OR "physical performance" OR

"exercise performance" OR endurance OR strength OR fatigue OR

recovery OR "muscle soreness" OR "muscle damage" OR

"muscle function" OR "skeletal muscle" OR "muscle mass" OR

"fatigue resistance" OR "running economy" OR

"time to exhaustion" OR "oxygen uptake" OR VO2 OR

"perceived exertion" OR "training adaptation"):ti,ab,kw

#8 #4 OR #5 OR #6 OR #7

#9 #3 AND #8

Strategy 6. ClinicalTrials.gov

Date of final search: 30 September 2026.

The registry was searched for interventional studies using combinations of olive-derived intervention terms and exercise-related terms. No restriction on recruitment status was applied.

Intervention-related terms

"olive oil" OR "extra virgin olive oil" OR EVOO OR

"Olea europaea" OR "olive fruit" OR "olive fruit extract" OR

"olive fruit water" OR "olive leaf" OR "olive leaf extract" OR

hydroxytyrosol OR oleuropein OR oleocanthal OR oleacein OR

"maslinic acid" OR OliPhenolia

Exercise-related terms

exercise OR sport OR athlete OR training OR

"physical activity" OR "physical performance" OR

"exercise performance" OR endurance OR strength OR fatigue OR

recovery OR "muscle soreness" OR "muscle damage" OR

"muscle function" OR "skeletal muscle" OR

"fatigue resistance" OR "running economy" OR

"time to exhaustion" OR "oxygen uptake" OR

"perceived exertion"

Strategy 7. WHO International Clinical Trials Registry Platform (ICTRP)

Date of final search: 30 September 2026.

("olive oil" OR "extra virgin olive oil" OR EVOO OR

"Olea europaea" OR "olive fruit" OR "olive fruit extract" OR

"olive fruit water" OR "olive leaf" OR "olive leaf extract" OR

hydroxytyrosol OR oleuropein OR oleocanthal OR oleacein OR

"maslinic acid" OR OliPhenolia)

AND

(exercise OR sport OR athlete OR training OR

"physical activity" OR "physical performance" OR

"exercise performance" OR endurance OR strength OR fatigue OR

recovery OR "muscle soreness" OR "muscle damage" OR

"muscle function" OR "skeletal muscle" OR

"fatigue resistance")

Note. Abbreviations: CENTRAL, Cochrane Central Register of Controlled Trials; EVOO, extra virgin olive oil; ICTRP, International Clinical Trials Registry Platform; MeSH, Medical Subject Headings; WHO, World Health Organization.
