# Supplementary material for: Olive-Derived Nutritional Interventions in Exercise: Effects on Performance, Fatigue, Recovery, and Muscle Adaptation—A Systematic Review
Source: Nutrients. 2026 Nov 7;18(21):3699. doi: 10.3390/nu18213699 (PMC13765110; doi:10.3390/nu18213699)
Supplement: Supplementary file 1 [file nutrients-18-03699-s001.zip › Supplementary Table S2.pdf]

Supplementary Table S2. Retrospective Summary of Review Methods and Synthesis Decisions

Purpose: To document the methodological procedures and synthesis decisions used in this systematic review and clarify their retrospective reporting in the absence of prospective registration or a published review protocol.

Methodological component	Description and clarification
**Review objective**	To evaluate randomized controlled evidence on olive-derived nutritional interventions in relation to exercise performance, exercise-induced fatigue, post-exercise recovery, skeletal-muscle function, and training adaptation in humans.
**Protocol and registration**	The review was not prospectively registered, and no formal dated review protocol was published or archived. This supplementary table was prepared retrospectively during manuscript revision and does not constitute a prospective protocol.
**Eligibility criteria**	Eligibility was defined using the Population, Intervention, Comparator, Outcomes, and Study design (PICOS) framework, as described in Section 2.2. Eligible studies were randomized controlled human trials evaluating orally administered olive-derived interventions in healthy participants undergoing exercise, training, or physical-function assessment. Although the criteria are described as established before study selection, their prospective specification cannot be independently verified against a registered protocol.
**Information sources and search strategy**	Searches covered MEDLINE/PubMed, Scopus, Web of Science Core Collection, SPORTDiscus, CENTRAL, ClinicalTrials.gov, and WHO ICTRP, from inception to 30 September 2026. Reference-list screening and forward citation searching were also undertaken. Complete database-specific search strategies and retrieval counts are reported in Supplementary Table S1.
**Study selection and verification**	Initial title and abstract screening and full-text eligibility assessment were conducted by the author. Full-text eligibility decisions, including exclusions and included reports, were subsequently checked by a second reviewer. Independent duplicate screening of all titles and abstracts was not performed.
**Data extraction and verification**	Study characteristics, intervention and comparator details, exercise protocols, outcome measures, numerical findings, and other relevant information were extracted by the author using a standardized form. Extracted information was subsequently checked against the original study reports by a second reviewer.
**Unit of analysis**	The independent randomized controlled trial was the unit of analysis. Eleven reports representing 10 independent trials were included. Two publications arising from the same randomized trial (NCT04959006) were consolidated to avoid double-counting participants while retaining complementary outcomes.
**Outcome classification**	Findings were organized into exercise performance, physiological efficiency, perceptual fatigue and exertion, post-exercise recovery and muscle soreness, and skeletal-muscle function and adaptation. Exercise-related inflammatory, oxidative, immune, metabolic, mitochondrial, and molecular responses were treated as secondary outcomes. These classifications are described in Section 2.2.4; no independently verifiable prospective outcome hierarchy was available.
**Risk-of-bias assessment**	Risk of bias was assessed using Cochrane RoB 2, applying the appropriate tools for parallel-group and crossover randomized trials. One selected principal

	review-relevant result was assessed per independent trial. Initial judgments were reviewed and corroborated by a second reviewer. The resulting judgments were outcome-specific and should not be generalized to other outcomes reported within the same trial.
**Quantitative synthesis**	A meta-analysis was not undertaken because the included studies differed substantially in intervention composition, dosage, supplementation duration, participant characteristics, exercise protocols, and outcome definitions. The decision not to pool results was documented in the Methods, but its prospective specification cannot be verified in the absence of a dated protocol.
**Narrative synthesis**	A structured synthesis without meta-analysis was conducted using the SWiM reporting framework. Findings were grouped by olive-derived intervention class and outcome domain. Statistically significant and nonsignificant findings were considered together, without assuming a common effect across chemically distinct preparations.
**Certainty-of-evidence assessment**	The GRADE approach was introduced during manuscript revision as a post hoc methodological addition. Certainty was considered by intervention type and outcome, taking account of risk of bias, inconsistency, indirectness, imprecision, and publication bias. This assessment was not prospectively specified.
**Changes from prospectively planned analyses**	Because no formal dated protocol or registered analysis plan exists, deviations between prospectively planned and reported analyses cannot be reliably established. The addition of the GRADE assessment during manuscript revision is explicitly identified as a post hoc change.
**Methodological limitations**	The absence of prospective registration and independent duplicate title and abstract screening limits methodological transparency and may increase the risk of selection errors. Independent checking of full-text eligibility, extracted data, and risk-of-bias judgments provides additional verification but does not eliminate these limitations.

Note: This table is a retrospective transparency document describing the review procedures and synthesis decisions. It should not be interpreted as evidence of prospective protocol registration or as retrospective confirmation that all reported outcomes and analyses were prespecified.
